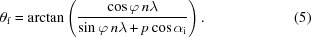# Grazing-incidence small-angle X-ray scattering (GISAXS) on small periodic targets using large beams. Erratum

**DOI:** 10.1107/S2052252518008497

**Published:** 2018-06-22

**Authors:** Mika Pflüger, Victor Soltwisch, Jürgen Probst, Frank Scholze, Michael Krumrey

**Affiliations:** a Physikalisch-Technische Bundesanstalt (PTB), Abbestraße 2-12, 10587 Berlin, Germany; b Helmholtz-Zentrum Berlin (HZB), Albert-Einstein-Straße 15, 12489 Berlin, Germany

**Keywords:** grazing-incidence small-angle X-ray scattering, GISAXS, beam footprint, lithographic inspection, gratings

## Abstract

An error in the paper by Pflüger, Soltwisch, Probst, Scholze & Krumrey [*IUCrJ* (2017), 431–438] is corrected.

## Abstract 2

An error in the article by Pflüger, Soltwisch, Probst, Scholze & Krumrey [*IUCrJ* (2017), 431–438] is corrected.

In the article by Pflüger *et al.* (2017 ▸), there is an error in equation (5) (§2, p. 433). The arcsin function should be arctan.

The corrected equation thus reads