# Supplementary material for: Grazing-incidence small-angle X-ray scattering (GISAXS) on small periodic targets using large beams
Source: IUCrJ. 2017 May 24;4(Pt 4):431–8. doi: 10.1107/S2052252517006297 (PMC5571806; doi:10.1107/S2052252517006297)
Supplement: Supplementary file 1 [file m-04-00431-sup1.pdf]

IUCrJ

Volume 4 (2017)

Supporting information for article:

Grazing-incidence small-angle X-ray scattering (GISAXS) on small periodic targets using large beams

Mika Pflüger, Victor Soltwisch, Jürgen Probst, Frank Scholze and Michael Krumrey

Supporting Information

Grazing Incidence Small Angle X-Ray Scattering (GISAXS) on Small Periodic Targets Using Large Beams

Mika Pflüger Victor Soltwisch Jürgen Probst
Frank Scholze Michael Krumrey

1 Background Correction

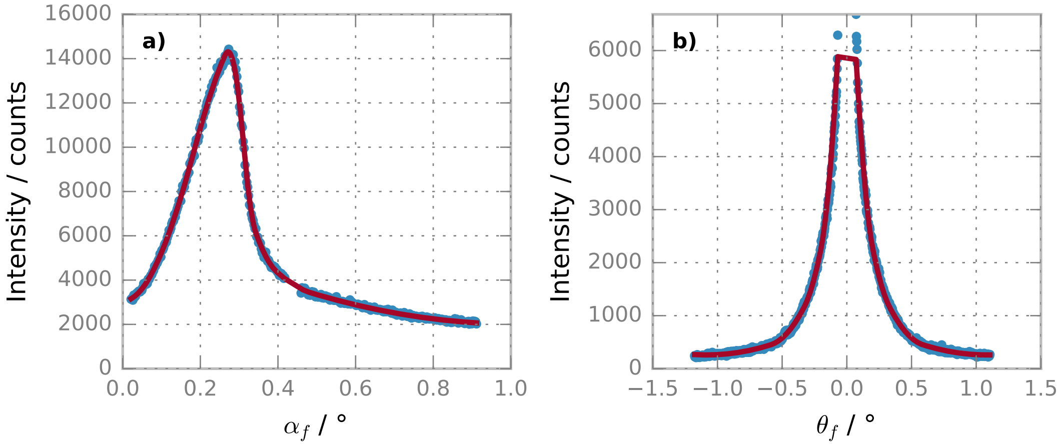

Figure 1: Background cuts (blue dots) and corresponding fits (red line). **a)** vertical cut and fitted smooth B-spline of degree 2. **b)** horizontal cut and fitted polynomial of degree 4.

In order to extract the scattering of the targets only, the background B was fitted for each measurement, assuming that the background B can be factorized to $B(\alpha_f, \theta_f) = A(\alpha_f) \cdot T(\theta_f)$. This factorization is motivated by the assumption that $T(\theta_f)$ depends mainly on the correlations of the roughness of the substrate in x- and y-direction, which in small-angle approximation does not depend on α_f. For the function $A(\alpha_f)$, a smooth B-spline approximation of degree 2 was used to closely follow the scattering of the background around the critical angle of total external reflection α_c of the substrate (see fig. 1 a)). In order to only fit the substrate contribution, a cut along α_f was taken between the first and second grating diffraction orders. For the function $T(\theta_f)$, a polynomial of degree 4 was fitted to a cut along θ_f at $\alpha_f > 0.8°$, i.e.

above the sample scattering features (see fig. 1 b)). The resulting smooth background was subtracted from the GISAXS measurement, yielding the scattering from the target only (fig. 2).

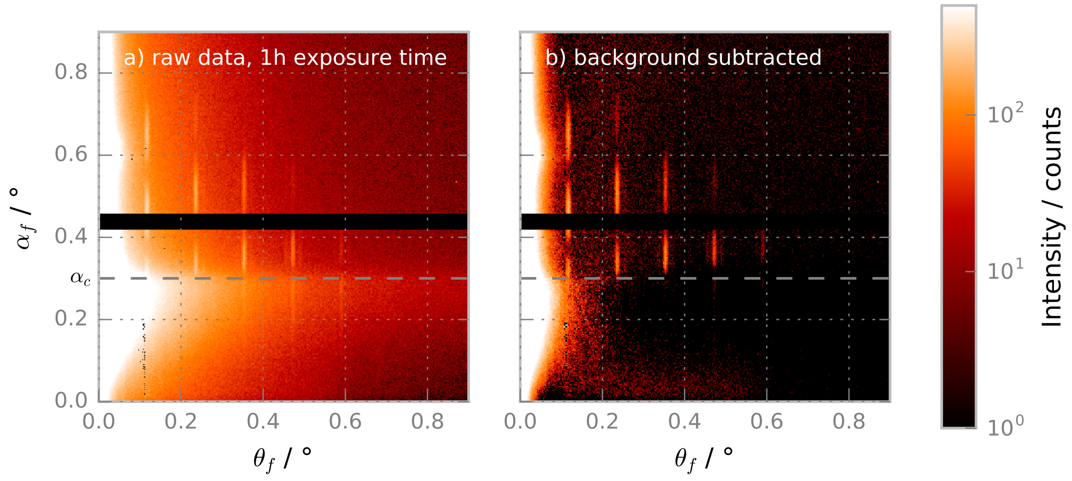

Figure 2: GISAXS scattering of smallest target, **a)** raw data **b)** after background subtraction. The background subtraction works well above the critical angle of the substrate α_c, but fails below α_c.

2 Position of Grating Diffraction Orders in GISAXS in Sample Coordinates

2.1 Coordinate System and Ewald Sphere

We use a coordinate system where the x-y-plane is the sample plane, with the x-axis the intersection of the scattering plane with the sample plane and the y-axis perpendicular to the x-axis. The z-axis is the normal of the sample plane. The k-space is the reciprocal of the real space, with the corresponding axes in the same direction as the real axes. In this space, the wavevectors of the incoming beam $\boldsymbol{k}_i$ and the scattered beam $\boldsymbol{k}_f$ are

$$\boldsymbol{k}_i = k_0 \begin{pmatrix} \cos\alpha_i \\ 0 \\ -\sin\alpha_i \end{pmatrix} \tag{1}$$

$$\boldsymbol{k}_f = k_0 \begin{pmatrix} \cos\alpha_f \cos\theta_f \\ \cos\alpha_f \sin\theta_f \\ \sin\alpha_f \end{pmatrix} \tag{2}$$

$$k_0 = |\boldsymbol{k}_i| = |\boldsymbol{k}_f| = \frac{2\pi}{\lambda} \tag{3}$$

with the incident angle α_i, the angle between the sample plane and the scattered beam α_f and the angle between the projection of the scattered beam on the sample plane and the x-axis θ_f

as well as the incident wavelength λ.

We define the scattering vector $\boldsymbol{q} = \boldsymbol{k}_f - \boldsymbol{k}_i$, which expressed in angle coordinates is

$$\boldsymbol{q} = k_0 \begin{pmatrix} \cos\alpha_f \cos\theta_f - \cos\alpha_i \\ \cos\alpha_f \sin\theta_f \\ \sin\alpha_f + \sin\alpha_i \end{pmatrix}, \tag{4}$$

together with (3) we can write the equation for the Ewald sphere of elastic scattering

$$k_0 = |\boldsymbol{k}_f| = |\boldsymbol{q} + \boldsymbol{k}_i| \tag{5}$$

$$\Rightarrow k_0^2 = |\boldsymbol{q} + \boldsymbol{k}_i|^2 = (q_x + k_{i,x})^2 + (q_y + k_{i,y})^2 + (q_z + k_{i,z})^2$$

$$= (q_x + k_0 \cos\alpha_i)^2 + q_y^2 + (q_z - k_0 \sin\alpha_i)^2 \quad . \tag{6}$$

2.2 Perfectly Aligned Grating

The perfectly aligned grating has infinite grating lines parallel to the x-axis, which lie in the sample plane and are separated by the pitch p. The reciprocal space representation of the perfectly aligned grating comprises grating truncation rods (GTR), which are parallel to the q_z-axis in the q_z-q_y-plane and separated by $2\pi/p$ in q_y:

$$q_x = 0 \tag{7}$$

$$q_y = n 2\pi/p = k_0 \, n\lambda/p \tag{8}$$

with the grating diffraction order $n \in \mathbb{Z}$. The intersection of the Ewald sphere (6) with the GTR yields

$$k_0^2 = (0 + k_0 \cos\alpha_i)^2 + (n k_0 \lambda/p)^2 + (q_z - k_0 \sin\alpha_i)^2 \tag{9}$$

solving for q_z

$$(q_z - k_0 \sin\alpha_i)^2 = k_0^2 (1 - \cos^2\alpha_i) - (n k_0 \lambda/p)^2$$

$$= k_0^2 \left(\sin^2\alpha_i - (n\lambda/p)^2\right) \tag{10}$$

$$\Rightarrow q_z = k_0 \left(\sin\alpha_i \pm \sqrt{\sin^2\alpha_i - (n\lambda/p)^2}\right) \tag{11}$$

discarding the solution with the minus as it corresponds to reflections below the sample horizon

$$q_z = k_0 \left(\sin\alpha_i + \sqrt{\sin^2\alpha_i - (n\lambda/p)^2}\right) \quad . \tag{12}$$

To summarize:

$$\boldsymbol{q}_{\text{grating, aligned}} = k_0 \begin{pmatrix} 0 \\ n\lambda/p \\ \sin\alpha_i + \sqrt{\sin^2\alpha_i - (n\lambda/p)^2} \end{pmatrix} \quad . \tag{13}$$

To express the scattering in angle coordinates, we use (4), (7), (8) and (12), giving

$$q_z: \qquad \sin\alpha_f + \sin\alpha_i = \sin\alpha_i\left(1+\sqrt{1-\left(\frac{n\lambda}{p\sin\alpha_i}\right)^2}\right)$$

$$\Rightarrow \alpha_f = \arcsin\left(\sqrt{\sin^2\alpha_i - \left(\frac{n\lambda}{p}\right)^2}\right) \tag{14}$$

$$q_y: \qquad \cos\alpha_f \sin\theta_f = n\lambda/p$$

$$\Rightarrow \sin\theta_f = \frac{n\lambda/p}{\cos\alpha_f} \tag{15}$$

$$q_x: \qquad \cos\alpha_f\cos\theta_f - \cos\alpha_i = 0$$

$$\Rightarrow \cos\theta_f = \frac{\cos\alpha_i}{\cos\alpha_f} \tag{16}$$

$$\frac{q_y}{q_x}: \qquad \tan\theta_f = \frac{\sin\theta_f}{\cos\theta_f} = \frac{n\lambda/p}{\cos\alpha_f}\frac{\cos\alpha_f}{\cos\alpha_i}$$

$$\Rightarrow \theta_f = \arctan\left(\frac{n\lambda}{p\cos\alpha_i}\right) \tag{17}$$

2.3 Misaligned Grating

For the misaligned grating, the grating lines are rotated around the z-axis by φ, and thus the GTRs are also rotated around the k_z-axis by φ, giving the conditions

$$q_x = k_0 \sin\varphi\, n\lambda/p \tag{18}$$

$$q_y = k_0 \cos\varphi\, n\lambda/p \quad . \tag{19}$$

The intersection with the Ewald sphere (6) now yields

$$\begin{aligned} k_0^2 &= (k_0\sin\varphi\, n\lambda/p + k_0\cos\alpha_i)^2 + (k_0\cos\varphi\, n\lambda/p)^2 + (q_z - k_0\sin\alpha_i)^2 \\ &= k_0^2\left((\sin^2\varphi + \cos^2\varphi)(n\lambda/p)^2 + 2\sin\varphi\cos\alpha_i\, n\lambda/p + \cos^2\alpha_i\right) + (q_z - k_0\sin\alpha_i)^2 \end{aligned} \tag{20}$$

solving for q_z

$$\begin{aligned} (q_z - k_0\sin\alpha_i)^2 &= k_0^2\left(1-\cos^2\alpha_i - (n\lambda/p)^2 - 2\sin\varphi\cos\alpha_i\, n\lambda/p\right) \\ &= k_0^2\left(\sin^2\alpha_i - (n\lambda/p)^2 - 2\sin\varphi\cos\alpha_i\, n\lambda/p\right) \end{aligned} \tag{21}$$

$$\Rightarrow q_z = k_0\left(\sin\alpha_i \pm \sqrt{\sin^2\alpha_i - (n\lambda/p)^2 - 2\sin\varphi\cos\alpha_i\, n\lambda/p}\right) \tag{22}$$

discarding the solution with the minus as it corresponds to reflections below the sample horizon

$$q_z = k_0\left(\sin\alpha_i + \sqrt{\sin^2\alpha_i - (n\lambda/p)^2 - 2\sin\varphi\cos\alpha_i\, n\lambda/p}\right) \quad . \tag{23}$$

To summarize:

$$\boldsymbol{q}_{\text{grating}} = k_0 \begin{pmatrix} \sin\varphi\, n\lambda/p \\ \cos\varphi\, n\lambda/p \\ \sin\alpha_i + \sqrt{\sin^2\alpha_i - (n\lambda/p)^2 - 2\sin\varphi\cos\alpha_i\, n\lambda/p} \end{pmatrix} \quad . \tag{24}$$

To express the scattering in angle coordinates, we use (4), (18), (19) and (23), giving

$$q_z: \quad \sin\alpha_f + \sin\alpha_i = \sin\alpha_i + \sqrt{\sin^2\alpha_i - (n\lambda/p)^2 - 2\sin\varphi\cos\alpha_i\, n\lambda/p}$$

$$\Rightarrow \alpha_f = \arcsin\left(\sqrt{\sin^2\alpha_i - (n\lambda/p)^2 - 2\sin\varphi\cos\alpha_i\, n\lambda/p}\right) \tag{25}$$

$$q_y: \quad \cos\alpha_f \sin\theta_f = \cos\varphi\, n\lambda/p$$

$$\Rightarrow \sin\theta_f = \frac{\cos\varphi\, n\lambda/p}{\cos\alpha_f} \tag{26}$$

$$q_x: \quad \cos\alpha_f \cos\theta_f - \cos\alpha_i = \sin\varphi\, n\lambda/p$$

$$\Rightarrow \cos\theta_f = \frac{\sin\varphi\, n\lambda/p + \cos\alpha_i}{\cos\alpha_f} \tag{27}$$

$$\frac{q_y}{q_x}: \quad \tan\theta_f = \frac{\sin\theta_f}{\cos\theta_f} = \frac{\cos\varphi\, n\lambda/p}{\cos\alpha_f} \frac{\cos\alpha_f}{\sin\varphi\, n\lambda/p + \cos\alpha_i}$$

$$\Rightarrow \theta_f = \arctan\left(\frac{\cos\varphi\, n\lambda/p}{\sin\varphi\, n\lambda/p + \cos\alpha_i}\right) \quad . \tag{28}$$
