# Supplementary material for: Grazing-incidence small-angle X-ray scattering (GISAXS) on small periodic targets using large beams
Source: IUCrJ. 2017 May 24;4(Pt 4):431–8. doi: 10.1107/S2052252517006297 (PMC5571806; doi:10.1107/S2052252517006297)
Supplement: Supplementary file 3 [file m-04-00431-sup3.html]

–


+

 Once
 Loop
 Reflect
